# Comparison of MIBG uptake in the major salivary glands between Lewy body disease and progressive supranuclear palsy

**DOI:** 10.1016/j.prdoa.2024.100287

**Published:** 2024-11-20

**Authors:** Junya Ebina, Sunao Mizumura, Mari Shibukawa, Harumi Morioka, Junpei Nagasawa, Masaru Yanagihashi, Takehisa Hirayama, Nobutomo Ishii, Yukio Kobayashi, Akira Inaba, Satoshi Orimo, Osamu Kano

**Affiliations:** a Department of Neurology, Toho University Faculty of Medicine, Tokyo, Japan; b Department of Radiology, Toho University Faculty of Medicine, Tokyo, Japan; c Central Radiology Division, Department of Radiology, Toho University Omori Medical Center, Tokyo, Japan; d Department of Radiological Technology, Kanto Central Hospital, Tokyo, Japan; e Department of Neurology, Kanto Central Hospital, Tokyo, Japan; f Kamiyoga Setagaya Street Clinic, Tokyo, Japan

**Keywords:** Parkinson’s disease, Dementia with Lewy bodies, Progressive supranuclear palsy, MIBG scintigraphy, Major salivary glands

## Abstract

**Introduction:**

Cardiac sympathetic denervation is specific to Lewy body disease (LBD). In Parkinson’s disease (PD), sympathetic denervation in the major salivary glands (parotid glands [PG] and submandibular glands [SMG]) has been demonstrated by ^123^I-metaiodobenzylguanidine (MIBG) scintigraphy. We compared sympathetic denervation in the MSG between PD, dementia with Lewy bodies (DLB), and progressive supranuclear palsy (PSP).

**Methods:**

We recruited 81 patients with PD, 12 with DLB, 13 with PSP and 25 with control subjects. We evaluated MIBG uptake in the major salivary glands and heart using a quantitative semi-automatic method. We compared MIBG uptake between PD, DLB, and PSP patients and controls, and we evaluated disease sensitivity and specificity. We compared olfactory function with MIBG uptake between PD and PSP patients.

**Results:**

MIBG uptake in the PG and SMG in the delayed phase was significantly lower in PD and DLB patients than in PSP patients and controls. Conversely, MIBG uptake in the major salivary glands and heart was comparable between PD and DLB. Between LBD and non-LBD, MIBG uptake showed 56–100 % specificity in the PG, while it had 55.6–87.5 % sensitivity in the SMG. Between PD and PSP, MIBG uptake in the PG and SMG had higher disease specificity than olfactory function, while the sensitivity of SMG MIBG uptake was comparable to olfactory function.

**Conclusion:**

PD and DLB patients showed lower MIBG uptake in the major salivary glands than PSP patients, especially in the delayed phase. MIBG uptake in the major salivary glands may differentiate PD from hyposmic PSP.

## 1 Introduction

Parkinson’s disease (PD) and dementia with Lewy bodies (DLB) are classified as Lewy body disease (LBD) and are pathologically included in synucleinopathies. For a diagnosis of PD, parkinsonism defined by the presence of bradykinesia in combination with rigidity and rest tremor is a mandatory feature [1]. However, non-motor features such as olfactory disturbance, rapid eye movement sleep behavior disorder, and cardiac sympathetic denervation that occasionally precede the motor features are also crucial [1], [2], and these features are generally shared with DLB. As a specific clinical characteristic, LBD shows cardiac sympathetic denervation on ^123^I-metaiodobenzylguanidine (MIBG) scintigraphy, which is included in the Movement Disorder Society (MDS) criteria for PD [1] and the diagnostic criteria for DLB [3].

Multiple system atrophy and progressive supranuclear palsy (PSP), which are both classified as atypical parkinsonism, share similar clinical features with LBD, especially in the early stage of the disease. The pathological background of multiple system atrophy is glial cytoplasmic inclusions, consisting of α-synuclein like LBD. PSP is classified as a four-repeat tauopathy, and tau accumulates in the subcortical area, which is associated with cortical neuronal dysfunction, as demonstrated by a recent positron emission tomography study [4].

Cardiac sympathetic denervation can differentiate LBD from other similar syndromes such as multiple system atrophy and PSP [5], [6], [7], [8]. What is important is that the reduced cardiac MIBG uptake in LBD is strictly associated with cardiac sympathetic denervation and the presence of α-synuclein-positive Lewy body pathology [9], [10]. However, α-synuclein-positive Lewy body pathology spreads to various peripheral organs such as the major salivary glands [11], [12], [13] as well as the heart in LBD.

Sympathetic denervation of the major salivary glands in patients with PD has recently been introduced as a new radiological feature [14], [15], [16], [17]. We identified sympathetic denervation in the major salivary glands on MIBG scintigraphy in patients with PD by using a novel quantitative semi-automatic method [18], [19]. Therefore, we consider that the investigation of peripheral sympathetic denervation is crucial for understanding the progression of LBD. In the present study, we aimed to compare MIBG uptake in the major salivary glands (parotid glands [PG] and submandibular glands [SMG]) and to evaluate its sensitivity and specificity to differentiate between LBD and PSP.

## 2 Methods

### 2.1 Participants

We recruited patients with PD, DLB, and PSP and non-parkinsonian controls who underwent MIBG scintigraphy at Toho University Omori Medical Center between October 2020 and June 2024. We diagnosed patients with PD according to the current MDS criteria and early PD criteria [1], [20], DLB according to the current diagnostic criteria [2], and PSP according to the previous and current criteria [21], [22]. All patients with PD fulfilled the clinically probable or established criteria and early established criteria. All patients with DLB fulfilled the probable criteria. By contrast, all patients with PSP fulfilled the probable criteria in the MDS criteria. Of note, we determined the clinical phenotype of PSP according to the MDS criteria. We recruited non-parkinsonian controls who did not have dementia, neurodegenerative parkinsonism, secondary parkinsonism, and psychiatric disorders, referring to the medical records.

We excluded participants as follows: 1) those who were administered reserpine, tri-/tetracyclic depressants, opioids, other neuroleptics, and cardiovascular drugs that potentially have an effect on MIBG uptake, referring to a previous review in which the MIBG inhibitory was regarded as moderate to strong [23]; 2) those who were administered selegiline, which also affects MIBG uptake; 3) those who had diabetes; and 4) those who had syndromes and organic diseases and took medications that have a potential effect on the secretion of saliva, such as Sjögren's syndrome, sialolithiasis and anticholinergic*.*

This study was approved by the Ethics Committee of Toho University Omori Medical Center, and we obtained written informed consent from all participants in the present study (M20101, M22232). The study was conducted in compliance with the Declaration of Helsinki.

### 2.2 Clinical evaluations

We collected the following clinical data from all participants: age at examination, duration of illness, and Mini-Mental State Examination for cognitive function. To evaluate hyposmia in PD and PSP, we used the Odor Stick Identification Test for Japanese (OSIT-J), which is comprised of 12 items that all are common odors in Japan. We defined severe hyposmia as a score of ≤4 for the detection of the 12 odors in the OSIT-J. We used the Hoehn-Yahr (H-Y) stage and MDS-Unified Parkinson’s Disease Rating Scale (MDS-UPDRS) Parts I, II, and III in patients with PD and PSP to assess disease severity in non-motor, activities of daily living, and motor aspects of the disease. Due to a lack of data, we did not include the OSIT-J score, H-Y stage, and MDS-UPDRS in patients with DLB.

### 2.3 MIBG scintigraphy scanning protocol and analysis

We acquired head and chest planar images of the early and delayed phases after injection of 111 MBq ^123^I-MIBG (MyoMIBG®; PDRadiopharma, Inc., Tokyo, Japan) at 20 and 240 min using two different single photon emission computed tomography (SPECT) scanners because of the replacement of the SPECT scanner at Toho University Omori Medical Center. Between October 2020 and November 2021, we performed MIBG scintigraphy using an Infinia GP3 gamma camera (GE Healthcare, Piscataway, NJ, USA) and an ELEGP collimator (GE Healthcare) (SPECT scanner A), and between December 2021 and June 2024, using a Symbia Intevo Bold gamma camera (Siemens, Erlangen, Germany) and an LMEGP collimator (Siemens) (SPECT scanner B). The matrix size was 256 × 256, and the head and chest acquisition time was 5 min, respectively.

We performed imaging analysis using smart MIBG software (PDRadiopharma, Inc.). The data analysis scheme has been described previously [18]. In brief, we analyzed MIBG uptake in the PG, SMG, and heart overlaying the chest and head planar images in the early and delayed phases, respectively. Then, we set the regions of interest at the left and right PG, SMG, and heart, fixing the appropriate location to analyze between the early and delayed phase planar images, semi-automatically. We calculated MIBG uptake as the ratio of the PG/mediastinum (P/M), SMG/mediastinum (S/M), and heart/mediastinum (H/M). Additionally, since we used different SPECT machines because of machine replacement, we calculated conversion coefficients to preserve data continuity between the machines [24], [25].

### 2.4 Statistical analysis

We performed statistical analysis using IBM SPSS Statistics Ver.29 (IBM Japan, Tokyo, Japan). Each graph in the present study was made by GraphPad Prism 10 (MDF, Tokyo, Japan). We analyzed the clinical characteristics and MIBG uptake in the PG, SMG, and heart in each group using the Kruskal-Wallis test, Mann-Whitney *U* test, and chi-square test, as appropriate. We used a *post-hoc* Dunn’s test in the Kruskal-Wallis test.

Furthermore, we evaluated receiver operating characteristic (ROC) curves to determine the appropriate cut-off values for the PG, SMG, and heart by the maximum value of the Youden index. After setting the optimal cut-off values in each area, we evaluated disease sensitivity and specificity of MIBG uptake between PD, DLB, and PSP and between PD, DLB, and controls, respectively. Additionally, we assessed the sensitivity and specificity of severe hyposmia (OSIT-J score ≤ 4) in comparison with MIBG uptake as another method to differentiate between PD and PSP. We also evaluated MIBG uptake in the major salivary glands and heart and the ROC curves between LBD and PSP, between LBD and controls, and between LBD and non-LBD.

Furthermore, we examined the correlations between MIBG uptake and clinical features in PD, DLB, and PSP patients and controls. We performed correlation analysis using Pearson’s coefficient or Spearman’s rank correlation coefficient, considering Gaussian distributions. Since the major salivary glands are a left–right paired organ, we used the average value for correlation analysis between MIBG uptake in the PG and SMG, clinical characteristics, and cardiac MIBG uptake. We additionally used a false discovery rate correction for correlation analysis to adjust for multiple comparisons. We set statistical significance at p < 0.05.

## 3 Results

### 3.1 Demographics and clinical characteristics

The demographics and clinical characteristics of the participants are displayed in Table 1. We recruited the following sex-duration of illness-matched patients: 81 with PD, 12 with DLB, and 13 with PSP (5 with PSP-Richardson syndrome, 6 with PSP-parkinsonism, and 2 with PSP-progressive gait freezing); we also recruited 25 non-parkinsonian controls (21 with gait disturbance associated with orthopedic problems, geriatric syndrome and aging, 2 with motor neuron disease, and 2 with dystonia). Patients with PD tended to show more severe hyposmia than those with PSP, but without a significant difference. By contrast, patients with PSP showed more severe impairment of the activities of daily living and motor function than those with PD. PG, SMG, and cardiac MIBG uptake was significantly different in group comparisons (Table 1).

**Table 1:** Demographics and clinical characteristics of the participants.

	PD	DLB	PSP	Controls	p-value
Number (Female)	81 (44)	12 (7)	13 (4)	25 (9)	0.191
Age (years)	71.7 ± 9.2	80.0 ± 4.0	75.9 ± 6.8	68.7 ± 14.4	**0.005**
SPECT A/B	33/48	7/5	6/7	5/20	0.108
MMSE	26.6 ± 4.6	19.2 ± 7.0	25.9 ± 3.5	27.4 ± 1.8	**<0.001**
Duration of illness (years)	3.2 ± 3.1	2.4 ± 2.8	3.8 ± 2.4	N.A.	0.100
OSIT-J score	3.5 ± 2.8	N.A.	4.7 ± 3.3	N.A.	0.178
Severe hyposmia (%)	52 (57.8 %)	N.A.	5 (38.5 %)	N.A.	0.078
H-Y stage	2.4 ± 0.7	N.A.	3.2 ± 0.8	N.A.	**0.001**
MDS-UPDRS I	10.1 ± 6.1	N.A.	9.8 ± 4.5	N.A.	0.673
MDS-UPDRS II	11.3 ± 8.8	N.A.	20.2 ± 9.8	N.A.	**<0.001**
MDS-UPDRS III	29.6 ± 16.6	N.A.	39.3 ± 15.6	N.A.	**0.041**
Early P/M	1.12 ± 0.38	1.08 ± 0.29	1.32 ± 0.24	1.42 ± 0.33	**<0.001**
Delayed P/M	1.65 ± 0.49	1.63 ± 0.45	2.00 ± 0.36	2.05 ± 0.42	**<0.001**
Early S/M	1.34 ± 0.28	1.23 ± 0.25	1.46 ± 0.35	1.64 ± 0.28	**<0.001**
Delayed S/M	1.70 ± 0.30	1.62 ± 0.32	1.93 ± 0.33	2.06 ± 0.29	**<0.001**
Early H/M	2.09 ± 0.55	1.85 ± 0.45	2.85 ± 0.31	3.01 ± 0.40	**<0.001**
Delayed H/M	1.74 ± 0.63	1.57 ± 0.53	2.73 ± 0.48	3.01 ± 0.62	**<0.001**

DLB: dementia with Lewy bodies, H/M: heart/mediastinum ratio, H-Y: Hoehn-Yahr, MDS-UPDRS: Movement Disorder Society-Unified Parkinson’s Disease Rating Scale, MMSE: Mini-Mental State Examination, N.A.: not analyzed, OSIT-J: Odor Stick Identification Test for Japanese, PD: Parkinson’s disease, P/M: parotid gland/mediastinum ratio, PSP: progressive supranuclear palsy, S/M: submandibular gland/mediastinum ratio, SPECT: single photon emission computed tomography.

### 3.2 Comparisons of MIBG uptake in the major salivary glands and heart

**Fig. 1:**
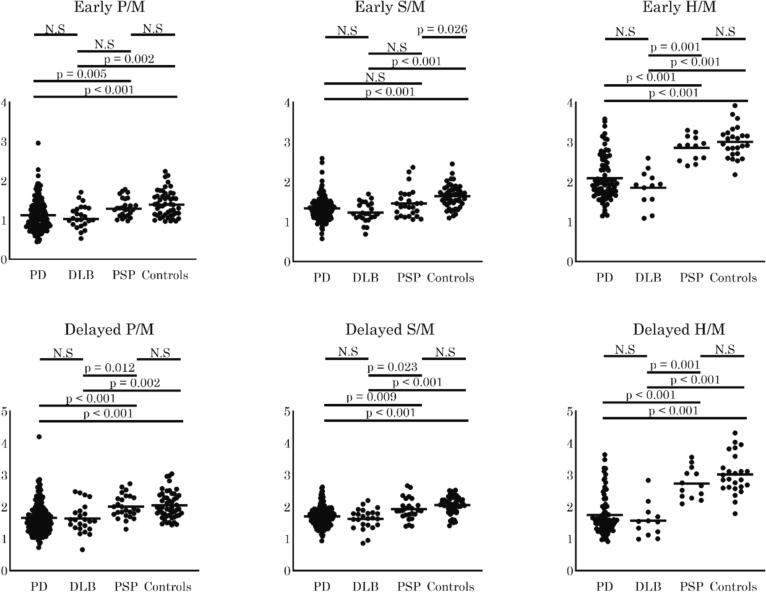
Comparisons of ^123^I-metaiodobenzylguanidine (MIBG) uptake among the groups. The outlines of comparisons of MIBG uptake in the parotid glands (PG), submandibular glands (SMG), and heart between Parkinson’s disease (PD), dementia with Lewy bodies (DLB), and progressive supranuclear palsy (PSP) patients and controls are shown. Each group’s horizontal bar indicates the average. H/M: heart/mediastinum ratio, P/M: parotid gland/mediastinum ratio, S/M: SMG/mediastinum ratio.

The outlines for the comparisons of MIBG uptake are shown in Fig. 1. Between PD and PSP, we found significantly reduced MIBG uptake in the PG, SMG, and heart, except for early phase MIBG uptake in the SMG. By contrast, there was a significant reduction of early and delayed MIBG uptake in the major salivary glands and heart in PD compared to controls (Fig. 1).

Between DLB and PSP, delayed MIBG uptake in the PG and SMG and both early and delayed cardiac MIBG uptake were significantly lower in DLB than in PSP. By contrast, between DLB and controls, all MIBG uptake in the major salivary glands and heart was lower in DLB than in controls (Fig. 1).

Between PD and DLB, there was no significant difference in MIBG uptake in the major salivary glands and heart. Furthermore, there was no significant difference in MIBG uptake in the major salivary glands and heart between PSP and controls; however, early phase MIBG uptake in the SMG was lower in patients with PSP (Fig. 1).

Between LBD and PSP, there were significant differences in MIBG uptake, except for early S/M uptake. By contrast, between LBD and controls, there were significant differences in MIBG uptake in all examined areas (Supplementary Fig. 1).

### 3.3 ROC curves between PD, DLB, PSP, and controls

**Fig. 2:**
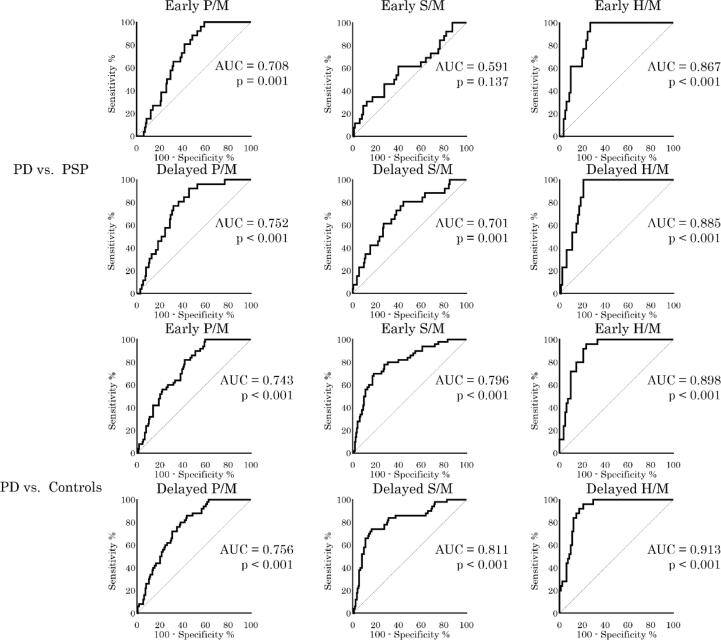

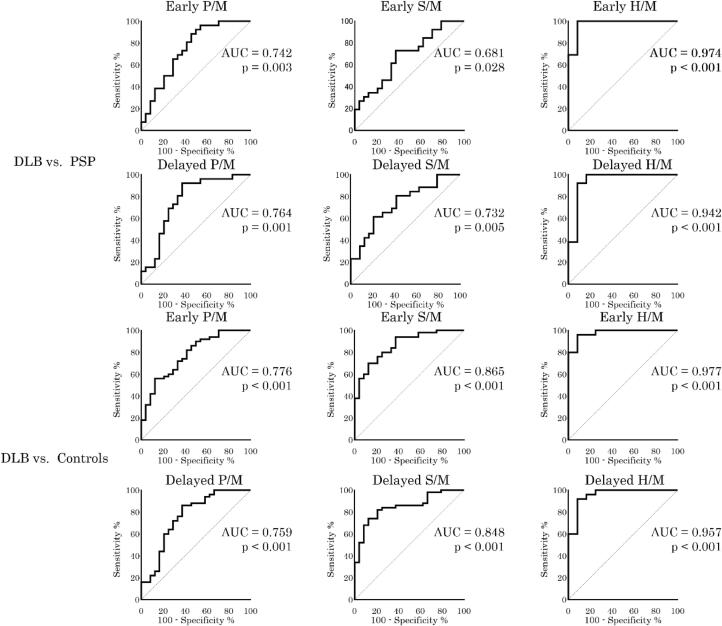
Receiver operating characteristic (ROC) curves between Parkinson’s disease (PD), dementia with Lewy bodies (DLB), and progressive supranuclear palsy (PSP) patients and controls. The ROC curves between PD and non-LBD and between DLB and non-LBD are shown. The area under the curve (AUC) and p-value are indicated in the ROC curves. Significant differences are found, except in early phase S/M between PD and PSP. H/M: heart/mediastinum ratio, P/M: parotid gland/mediastinum ratio, S/M: submandibular gland/mediastinum ratio.

We evaluated ROC curves between LBD and the other groups. In PD, there were significant differences in all comparisons, except for the early S/M ratio between PD and PSP. By contrast, in DLB, there were significant differences in all groups. The schemes for ROC curves with area under the curve are shown in Fig. 2. Additionally, the results between LBD and the other groups are shown in Supplementary Fig. 2.

### 3.4 Sensitivity and specificity of MIBG uptake and olfactory disturbance

Since we did not find a significant difference in the early S/M ratio on ROC curves between PD and PSP, we excluded this from the analysis. The overall results are listed in Table 2. Sensitivity was higher in the SMG (55.6–87.5 %) than in the PG (40.1–87.5 %) between LBD and the other groups. Conversely, specificity was higher in the PG (56.0–100 %) than in the SMG (61.5–80.8 %) between LBD and the other groups. Furthermore, sensitivity was higher in DLB than in PD in the major salivary glands. By contrast, cardiac MIBG uptake showed 80–90 % sensitivity and 90–100 % specificity in LBD (Table 2).

**Table 2:** Sensitivity and specificity of ^123^I-metaiodobenzylguanidine uptake between LBD, PSP, and controls.

%	PD vs. PSP		PD vs. Controls		DLB vs. PSP		DLB vs. Controls	
	Sensitivity	Specificity	Sensitivity	Specificity	Sensitivity	Specificity	Sensitivity	Specificity
Early P/M	40.7	100	40.1	100	54.2	88.5	87.5	56.0
Delayed P/M	54.3	92.3	56.2	86.0	62.5	92.3	62.5	86.0
Early S/M	N.A.	N.A.	81.5	70.0	62.5	73.1	87.5	70.0
Delayed S/M	55.6	80.8	83.3	74.0	79.2	61.5	87.5	74.0
Early H/M	72.8	100	76.5	96.0	91.7	100	91.7	96.0
Delayed H/M	79.0	100	79.0	96.0	91.7	92.3	91.7	92.0
Severe hyposmia	57.8	61.5	N.A.	N.A.	N.A.	N.A.	N.A.	N.A.

DLB: dementia with Lewy bodies, H/M: heart/mediastinum ratio, N.A.: not analyzed, PD: Parkinson’s disease, P/M: parotid gland/mediastinum ratio, PSP: progressive supranuclear palsy, S/M: submandibular gland/mediastinum ratio.

For the comparison of olfactory disturbance and MIBG uptake, when the cut-off was set at an OSIT-J score ≤ 4, specificity was higher for PG, SMG, and cardiac MIBG uptake. Conversely, sensitivity was almost comparable to SMG MIBG uptake, but lower than for PG MIBG uptake (Table 2). Disease sensitivity and specificity between LBD and the other groups are shown in Supplementary Table 1.

### 3.5 Correlations between MIBG uptake and clinical features

In PD and PSP, MIBG uptake in the PG and SMG were positively correlated with each other. By contrast, we did not find a similar correlation in DLB and controls. We did not find a correlation between MIBG uptake in the major salivary glands and heart in all groups. The overall results are shown in Supplementary Table 2.

## 4 Discussion

We demonstrated that the delayed phase of MIBG uptake in the PG and SMG was significantly reduced in patients with PD and DLB compared to PSP patients and controls using a novel quantitative semi-automatic method. Furthermore, we found the disease specificity of MIBG uptake in the major salivary glands was superior to olfactory disturbance between PD and PSP in the present study cohort.

Sympathetic denervation in the major salivary glands demonstrated by MIBG scintigraphy among patients with PD has been introduced as a new radiological marker [14], [15], [16], [17], [18], [19]. We found that MIBG uptake in the major salivary glands was lower in patients with PD than in PSP patients, as demonstrated previously [16]. Our findings possibly show the expansion of neurodegeneration outside the brain and a new radiological feature in addition to cardiac MIBG uptake in PD compared to PSP [5], [6], [7], [8]. Additionally, as a novel aspect, we found sympathetic denervation in the major salivary glands even in patients with DLB. This result probably reflects the pathologically similar spectrum of synucleinopathies.

Perhaps, although our study indicates the peripheral autonomic dysfunction in the SMG among patients with PD, it is still elucidated that MIBG uptake in the major salivary glands is radiopathologically associated with peripheral sympathetic denervation, as reported for the cardiac sympathetic denervation observed in PD [9], [10]. In fact, cardiac sympathetic denervation might be a specific feature compared to other peripheral organs in PD [26], although sympathetic denervation was found in the major salivary glands [26], [27]. However, we previously demonstrated that patients with PD who had dual sympathetic denervation (major salivary glands and heart) showed more advanced non-motor features such as severe hyposmia, rapid eye movement sleep behavior disorder, and autonomic dysfunction [19]. Furthermore, the recent radiological hypothesis has argued that the progression of PD begins in the gut before moving to the brain or begins in the central nervous system, introduced as the “body-first vs. brain-first theory” [28]. As observed in PD, we found similar results in patients with DLB, which might be consistent with the concept of the “body-first vs. body-first theory.” Therefore, we now consider that peripheral sympathetic denervation is a crucial factor for understanding the progression of LBD.

In the present study, we found that the severity of hyposmia was not significantly different between PD and PSP, although patients with PD showed a greater reduction of olfactory function. Previous studies have demonstrated that patients with PSP present with more severe hyposmia than controls [29], [30]. Conversely, previous studies have demonstrated that cardiac MIBG uptake is associated with olfactory function in PD [18], [19], [28]. Thus, the patients with PSP in the present study were not consistent with these findings. Furthermore, although patients with PSP showed more severe impairment of motor function than those with PD (the average H-Y stage of PSP patients was 3.8 and they had more severe motor features on the MDS-UPDRS Part III than those with PD), cardiac MIBG uptake was preserved in this group. Thus, the patients with PSP in the current study cohort had different clinical characteristics from those in a previous study in which all PD patients with an H-Y stage of ≥3 presented with reduced cardiac MIBG uptake [6]. Therefore, we assumed that shared factors such as mental and physical status potentially contribute to the lower olfactory function in patients with PSP. Although further investigations are needed, from our findings, when hyposmia is found in a patient with PSP, it might be helpful to examine MIBG uptake in the major salivary glands in addition to cardiac MIBG uptake as an extra option.

The present study has several limitations that should be discussed. First, the number of subjects was small, particularly in patients with DLB and PSP. The statistical power in the present study, perhaps, had an effect on the result between LBD and PSP for the early phase S/M ratio, and further investigations with a larger number of patients are warranted. Furthermore, we did not collect sufficient clinical data from the patients with DLB. We should also investigate patients with multiple system atrophy, which is an important form of atypical parkinsonism, in the future. Second, no pathologically confirmed cases were included. The diagnosis of PSP is still challenging because of its clinical varieties. Additionally, the radiopathological relationships between MIBG uptake and α-synuclein-positive Lewy pathology in the major salivary glands are unclear. Subsequently, sympathetic denervation in the major salivary glands potentially has different histopathological characteristics from that in the heart. Therefore, further investigations are needed to clarify the radiopathological relationships.

In conclusion, patients with PD and DLB showed lower MIBG uptake in the major salivary glands than patients with PSP, especially in the delayed phase, possibly demonstrating widespread sympathetic denervation in LBD in addition to that occurring in the heart. Furthermore, the evaluation of MIBG uptake in the major salivary glands might be helpful in differentiating PD from hyposmic PSP.

## CRediT authorship contribution statement

**Junya Ebina:** Writing – original draft, Visualization, Software, Project administration, Methodology, Investigation, Funding acquisition, Formal analysis, Data curation, Conceptualization. **Sunao Mizumura:** Writing – review & editing, Software, Methodology, Conceptualization. **Mari Shibukawa:** Writing – review & editing, Conceptualization. **Harumi Morioka:** Writing – review & editing, Conceptualization. **Junpei Nagasawa:** Writing – review & editing, Conceptualization. **Masaru Yanagihashi:** Writing – review & editing, Conceptualization. **Takehisa Hirayama:** Writing – review & editing, Conceptualization. **Nobutomo Ishii:** Writing – review & editing, Conceptualization. **Yukio Kobayashi:** Writing – review & editing, Conceptualization. **Akira Inaba:** Writing – review & editing, Conceptualization. **Satoshi Orimo:** Writing – review & editing, Supervision, Conceptualization. **Osamu Kano:** Writing – review & editing, Supervision, Conceptualization.

## Declaration of competing interest

The authors declare that they have no known competing financial interests or personal relationships that could have appeared to influence the work reported in this paper.
