# Supplementary material for: Comparison of MIBG uptake in the major salivary glands between Lewy body disease and progressive supranuclear palsy
Source: Clin Park Relat Disord. 2024 Nov 20;11:100287. doi: 10.1016/j.prdoa.2024.100287 (PMC11629250; doi:10.1016/j.prdoa.2024.100287)
Supplement: Supplementary Data 1 [file mmc1.docx]

Supplementary Table 1. Sensitivity and specificity of ^123^I-metaiodobenzylguanidine uptake between LBD, PSP, and controls

|  | LBD vs. PSP | | LBD vs. Controls | | LBD vs. Non-LBD | |
| --- | --- | --- | --- | --- | --- | --- |
| % | Sensitivity | Specificity | Sensitivity | Specificity | Sensitivity | Specificity |
| Early P/M | 44.1 | 96.2 | 58.1 | 82.0 | 82.3 | 57.9 |
| Delayed P/M | 55.4 | 92.3 | 57.0 | 86.0 | 83.9 | 63.2 |
| Early S/M | N.A. | N.A. | 82.3 | 70.0 | 58.1 | 81.6 |
| Delayed S/M | 55.9 | 80.8 | 83.9 | 74.0 | 55.4 | 88.2 |
| Early H/M | 75.3 | 100 | 78.5 | 96.0 | 75.3 | 97.4 |
| Delayed H/M | 79.6 | 100 | 83.9 | 92.0 | 79.6 | 97.4 |

H/M: heart/mediastinum ratio, N.A., not analyzed, LBD: Lewy body disease, P/M: parotid gland/mediastinum ratio, PSP: progressive supranuclear palsy, S/M: submandibular gland/mediastinum ratio.

Supplementary Table 2. Correlations between ^123^I-metaiodobenzylguanidine uptake and clinical characteristics

|  |  | PD | | | |
| --- | --- | --- | --- | --- | --- |
|  |  | Early P/M | Delayed P/M | Early H/M | Delayed H/M |
| Early P/M | r | N.A. | N.A. | -0.122 | N.A. |
|  | p | N.A. | N.A. | 0.278 | N.A. |
| Early S/M | r | **0.630** | N.A. | 0.020 | N.A. |
|  | p | **< 0.001** | N.A. | 0.858 | N.A. |
| Delayed P/M | r | N.A. | N.A. | N.A. | -0.208 |
|  | p | N.A. | N.A. | N.A. | 0.062 |
| Delayed S/M | r | N.A. | **0.525** | N.A. | 0.046 |
|  | p | N.A. | **< 0.001** | N.A. | 0.682 |

|  |  | PD | | | | | |
| --- | --- | --- | --- | --- | --- | --- | --- |
|  |  | Early  mean P/M | Delayed mean P/M | Early  mean S/M | Delayed  mean S/M | Early H/M | Delayed H/M |
| Age | r | 0.092 | 0.141 | -0.11 | -0.149 | -0.268 | **-0.326** |
|  | p | 0.412 | 0.209 | 0.326 | 0.185 | 0.016 | **0.003** |
| MMSE | r | 0.156 | 0.101 | 0.236 | 0.257 | 0.116 | 0.153 |
|  | p | 0.164 | 0.371 | 0.034 | 0.021 | 0.303 | 0.174 |
| Duration | r | 0.164 | 0.102 | -0.058 | -0.224 | -0.200 | **-0.266** |
|  | p | 0.143 | 0.366 | 0.609 | 0.044 | 0.073 | **0.016** |
| OSIT-J | r | -0.051 | -0.106 | 0.102 | 0.135 | 0.254 | **0.255** |
|  | p | 0.649 | 0.347 | 0.364 | 0.229 | 0.022 | **0.022** |
| H-Y stage | r | -0.023 | -0.007 | -0.064 | -0.095 | -0.174 | **-0.225** |
|  | p | 0.839 | 0.949 | 0.571 | 0.398 | 0.119 | **0.043** |
| MDS-UPDRS I | r | -0.157 | -0.136 | -0.284 | -0.196 | -0.286 | **-0.355** |
|  | p | 0.163 | 0.228 | 0.010 | 0.080 | 0.010 | **0.001** |
| MDS-UPDRS II | r | -0.019 | -0.002 | -0.193 | -0.195 | -0.274 | **-0.339** |
|  | p | 0.866 | 0.985 | 0.085 | 0.081 | 0.013 | **0.002** |
| MDS-UPDRS III | r | 0.037 | 0.023 | -0.114 | -0.134 | -0.234 | **-0.307** |
|  | p | 0.746 | 0.838 | 0.309 | 0.233 | 0.036 | **0.005** |

|  |  | DLB | | | |
| --- | --- | --- | --- | --- | --- |
|  |  | Early P/M | Delayed P/M | Early H/M | Delayed H/M |
| Early P/M | r | N.A. | N.A. | 0.080 | N.A. |
|  | p | N.A. | N.A. | 0.805 | N.A. |
| Early S/M | r | 0.378 | N.A. | 0.522 | N.A. |
|  | p | 0.069 | N.A. | 0.082 | N.A. |
| Delayed P/M | r | N.A. | N.A. | N.A. | -0.177 |
|  | p | N.A. | N.A. | N.A. | 0.583 |
| Delayed S/M | r | N.A. | 0.280 | N.A. | 0.005 |
|  | p | N.A. | 0.185 | N.A. | 0.988 |

|  | DLB | | | | | | |
| --- | --- | --- | --- | --- | --- | --- | --- |
|  |  | Early mean P/M | Delayed mean P/M | Early mean S/M | Delayed mean S/M | Early H/M | Delayed H/M |
| Age | r | 0.270 | 0.043 | -0.240 | -0.620 | -0.098 | -0.049 |
|  | p | 0.396 | 0.895 | 0.453 | 0.032 | 0.763 | 0.880 |
| MMSE | r | 0.225 | 0.112 | 0.060 | 0.211 | 0.274 | 0.127 |
|  | p | 0.482 | 0.728 | 0.854 | 0.511 | 0.388 | 0.695 |
| Duration | r | -0.079 | -0.211 | -0.498 | **-0.728** | -0.011 | 0.075 |
|  | p | 0.807 | 0.510 | 0.100 | **0.007** | 0.972 | 0.816 |

|  |  | PSP | | | |
| --- | --- | --- | --- | --- | --- |
|  |  | Early P/M | Delayed P/M | Early H/M | Delayed H/M |
| Early P/M | r | N.A. | N.A. | 0.056 | N.A. |
|  | p | N.A. | N.A. | 0.855 | N.A. |
| Early S/M | r | **0.391** | N.A. | 0.229 | N.A. |
|  | p | **0.048** | N.A. | 0.452 | N.A. |
| Delayed P/M | r | N.A. | N.A. | N.A. | 0.130 |
|  | p | N.A. | N.A. | N.A. | 0.671 |
| Delayed S/M | r | N.A. | **0.523** | N.A. | 0.234 |
|  | p | N.A. | **0.006** | N.A. | 0.441 |

|  |  | PSP | | | | | |
| --- | --- | --- | --- | --- | --- | --- | --- |
|  |  | Early  mean P/M | Delayed  mean P/M | Early  mean S/M | Delayed  mean S/M | Early H/M | Delayed H/M |
| Age | r | -0.208 | 0.125 | -0.100 | 0.051 | 0.368 | -0.050 |
|  | p | 0.495 | 0.685 | 0.744 | 0.868 | 0.216 | 0.872 |
| MMSE | r | 0.442 | 0.184 | 0.513 | 0.408 | -0.062 | 0.221 |
|  | p | 0.130 | 0.547 | 0.073 | 0.166 | 0.840 | 0.468 |
| Duration | r | -0.366 | -0.419 | 0.096 | -0.102 | -0.218 | -0.266 |
|  | p | 0.219 | 0.154 | 0.754 | 0.740 | 0.474 | 0.379 |
| OSIT-J | r | -0.162 | -0.510 | 0.011 | -0.318 | -0.020 | 0.257 |
|  | p | 0.597 | 0.075 | 0.972 | 0.289 | 0.950 | 0.397 |
| H-Y stage | r | -0.147 | -0.141 | -0.387 | -0.650 | -0.621 | -0.615 |
|  | p | 0.633 | 0.647 | 0.192 | 0.016 | 0.023 | 0.025 |
| MDS-UPDRS I | r | 0.267 | 0.333 | -0.244 | 0.038 | -0.130 | -0.044 |
|  | p | 0.378 | 0.266 | 0.421 | 0.902 | 0.671 | 0.887 |
| MDS-UPDRS II | r | -0.138 | -0.113 | -0.196 | -0.410 | -0.375 | -0.510 |
|  | p | 0.654 | 0.713 | 0.522 | 0.164 | 0.207 | 0.075 |
| MDS-UPDRS III | r | -0.271 | -0.207 | -0.071 | -0.048 | 0.087 | -0.466 |
|  | p | 0.370 | 0.498 | 0.818 | 0.876 | 0.779 | 0.109 |

|  |  | Controls | | | |
| --- | --- | --- | --- | --- | --- |
|  |  | Early P/M | Delayed P/M | Early H/M | Delayed H/M |
| Early P/M | r | N.A. | N.A. | -0.090 | N.A. |
|  | p | N.A. | N.A. | 0.669 | N.A. |
| Early S/M | r | 0.179 | N.A. | 0.365 | N.A. |
|  | p | 0.213 | N.A. | 0.073 | N.A. |
| Delayed P/M | r | N.A. | N.A. | N.A. | -0.388 |
|  | p | N.A. | N.A. | N.A. | 0.056 |
| Delayed S/M | r | N.A. | 0.221 | N.A. | 0.358 |
|  | p | N.A. | 0.123 | N.A. | 0.079 |

|  |  | Controls | | | | | |
| --- | --- | --- | --- | --- | --- | --- | --- |
|  |  | Early mean P/M | Delayed mean P/M | Early mean S/M | Delayed mean S/M | Early H/M | Delayed H/M |
| Age | r | 0.175 | 0.269 | -0.245 | -0.307 | -0.262 | -0.422 |
|  | p | 0.403 | 0.194 | 0.239 | 0.135 | 0.206 | 0.035 |
| MMSE | r | 0.187 | 0.196 | 0.243 | 0.322 | **0.517** | 0.441 |
|  | p | 0.372 | 0.348 | 0.242 | 0.116 | **0.008** | 0.027 |

Bold values indicate statistical significance even after false discovery correction. DLB: dementia with Lewy bodies, H/M: heart/mediastinum ratio, H-Y: Hoehn-Yahr, MDS-UPDRS: Movement Disorder Society-Unified Parkinson’s Disease Rating Scale, MMSE: Mini-Mental State Examination, N.A.: not analyzed, OSIT-J: Odor Stick Identification Test for Japanese, PD: Parkinson’s disease, P/M: parotid gland/mediastinum ratio, PSP: progressive supranuclear palsy, S/M: submandibular gland/mediastinum ratio.

Supplementary Figure 1. Comparisons of MIBG uptake among the groups


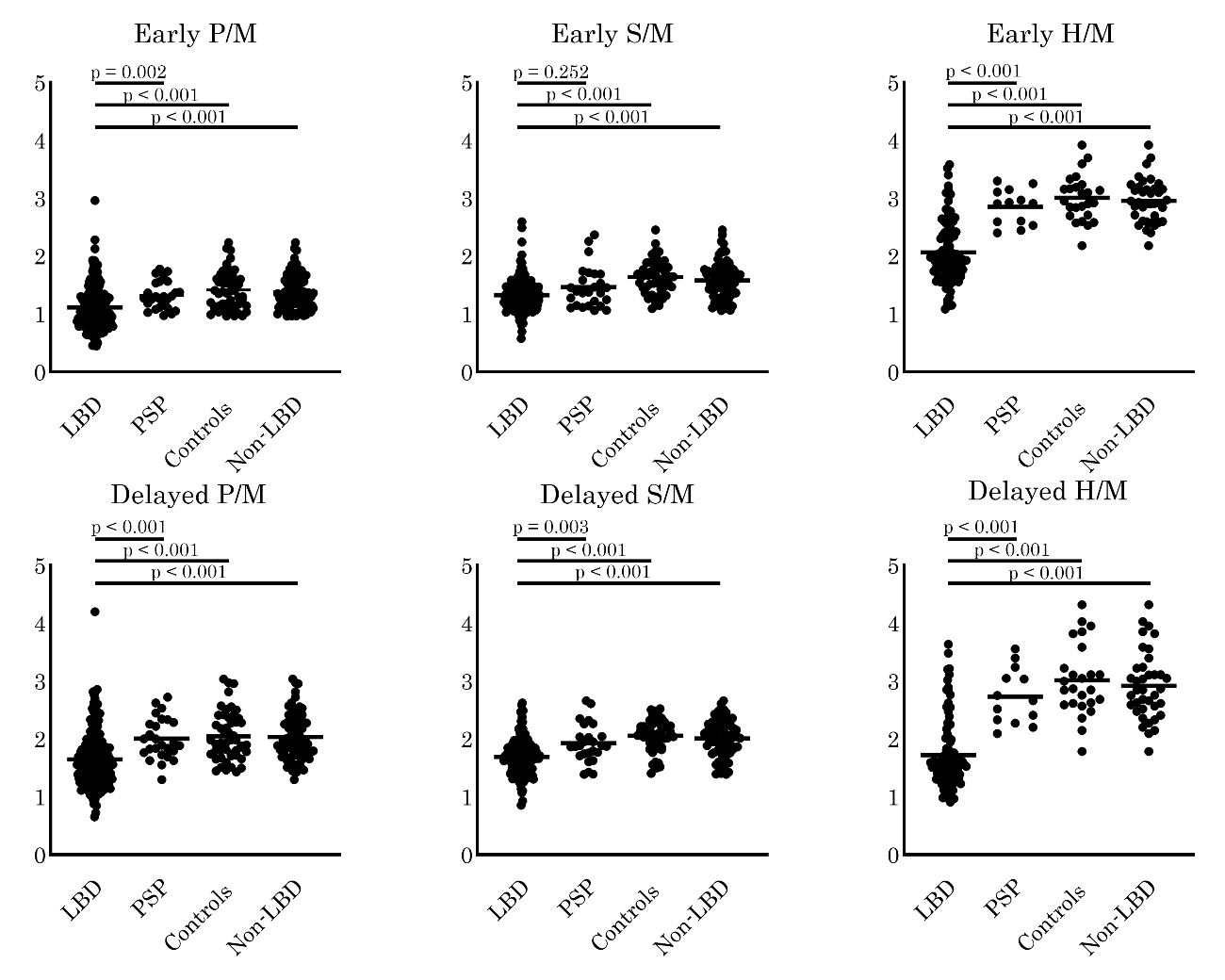


Figure Legend

Comparisons of MIBG uptake in the PG, SMG, and heart between LBD, PSP, and controls are shown. Each horizontal bar indicates the average. H/M: heart/mediastinum ratio, LBD: Lewy body disease, MIBG: ^123^I-metaiodobenzylguanidine, PG: parotid glands, P/M: parotid gland/mediastinum ratio, PSP: progressive supranuclear palsy, S/M: SMG/mediastinum ratio, SMG, submandibular glands.

Supplementary Figure 2. ROC curves between LBD, PSP, and controls


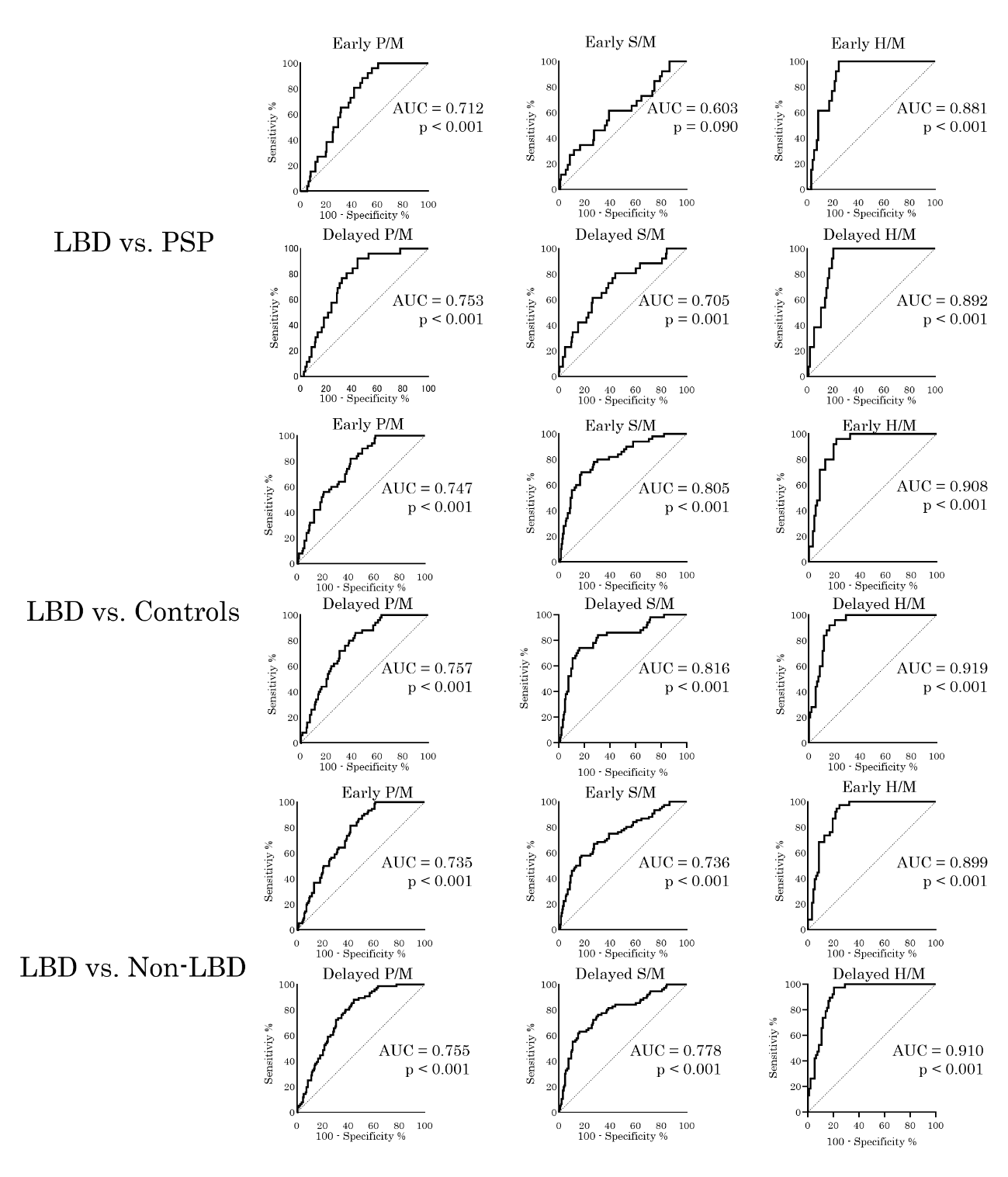


Figure Legend

ROC curves between LBD and PSP, LBD and controls, and LBD and non-LBD are shown. AUC and p-value are shown in the ROC curves. Significant differences are found, except in the early phase S/M ratio between LBD and PSP. AUC: area under the curve, H/M: heart/mediastinum ratio, LBD: Lewy body disease, P/M: parotid gland/mediastinum ratio, PSP: progressive supranuclear palsy, ROC: receiver operating characteristic, S/M: submandibular gland/mediastinum ratio.
